# Exosomal microRNAs as potential circulating biomarkers in gastrointestinal tract cancers: a systematic review protocol

**DOI:** 10.1186/s13643-017-0624-2

**Published:** 2017-11-17

**Authors:** Elmira Gheytanchi, Zahra Madjd, Leila Janani, Arezoo Rasti, Roya Ghods, Fatemeh Atyabi, Mohammad Hossein Asadi Lari, Sadegh Babashah

**Affiliations:** 1 Oncopathology Research Center, Iran University of Medical Sciences, Tehran, Iran; 2 Oncopathology Research Center, Department of Molecular Medicine, Faculty of Advanced Technologies in Medicine, Iran University of Medical Sciences, Tehran, Iran; 3 Department of Biostatistics, School of Public Health, Iran University of Medical Sciences, Tehran, Iran; 4 Department of Molecular Medicine, Faculty of Advanced Technologies in Medicine, Oncopathology Research Center, Iran University of Medical Sciences, Tehran, Iran; 5 Department of Pharmaceutics, Nanotechnology Research Center, Faculty of Pharmacy, Tehran University of Medical Sciences, Tehran, Iran; 6 Department of Cellular and Physiological Sciences, Faculty of Medicine, University of British Columbia (UBC), Vancouver, BC Canada; 7 Department of Molecular Genetics, Faculty of Biological Sciences, Tarbiat Modares University, Tehran, Iran

**Keywords:** Circulating, Exosomal microRNAs, Gastrointestinal tract, Cancer

## Abstract

**Background:**

Metastasis is the most frequent type of recurrence in gastrointestinal (GI) cancers, and there is an emerging potential for new diagnostic and therapeutic approaches, especially in the cases of metastatic GI carcinomas. The expression profiles of circulating exosomal microRNAs are of particular interest as novel non-invasive diagnostic and prognostic biomarkers for improved detection of GI cancers in body fluids, especially in the serum of patients with recurrent cancers. The aim of this study is to systematically review primary studies and identify the miRNA profiles of serum exosomes of GI cancers.

**Methods and design:**

This systematic review will be reported in line with the Preferred Reporting Items for Systematic Reviews and Meta-analyses (PRISMA) guidance. Relevant studies will be identified through a comprehensive search of the following main electronic databases: PubMed, Web of Science, Embase, Scopus, and Google Scholar, with no language restrictions (up to July 2017). Full copies of articles will be identified by a defined search strategy and will be considered for inclusion against pre-defined criteria. The quality assessment of the included studies will be performed by the Newcastle-Ottawa Scale (NOS). Data will be analyzed using Stata software V.12. Publication bias will be assessed by funnel plots, Beggs’ and Eggers’ tests. The levels of evidence for primary outcomes will be evaluated using the GRADE criteria.

**Discussion:**

The analysis of circulating exosomal miRNA profiles provides attractive screening and non-invasive diagnostic tools for the majority of solid tumors including GI cancers. There is limited information regarding the relationship between serum exosomal miRNA profiles and the pathological condition of patients with different GI cancers. Since there is no specific biomarker for GI cancers, we aim to suggest a number of circulating exosomal miRNA candidates as potential multifaceted GI cancer biomarkers for clinical utility.

**Systematic review registration:**

PROSPERO CRD42017057129

**Electronic supplementary material:**

The online version of this article (10.1186/s13643-017-0624-2) contains supplementary material, which is available to authorized users.

## Background

Gastrointestinal tract (GI) cancers represent a major challenge to healthcare systems given that they constitute 25% of cancer-related mortalities [1]. The incidence rate of GI cancers varies between developed and developing countries [2]. Among the 15 most frequent malignancies worldwide, one out of four new cancer cases and one out of three annual cancer-related deaths are attributed to GI cancers [3].

miRNAs are small non-coding RNA molecules consisting of approximately 22 nucleotides that control gene expression through targeting specific mRNAs bearing partially complementary target sequences for degradation and/or translational repression. miRNAs have been shown to be involved in regulating several cellular processes including cell differentiation, proliferation, and apoptosis in both normal and pathological conditions [4, 5]. miRNAs can act as either oncogenes or tumor suppressor genes in the networks specifically altered during cancer development and progression [4, 6, 7]. They are present as mobile genetic components for cell to cell communication or through the systemic circulation system within the body [4, 8].

Exosomes are nano-sized membrane vesicles, ranging from 30 to 100 nm in size, that are secreted by several cell types, including cancer cells, by exocytosis and can be isolated from different body fluids and malignant ascites [1, 4, 9]. Exosome content is composed of unique miRNAs, mRNA, DNA, lipids, and proteins that indicate the genetic information of their parental cells [10]. Exosomes play an important role in transferring bioactive oncogenic cargo to non-transformed recipient cells which cause tumor invasion, drug resistance, metastasis, and modulation of cancer metabolism [1, 11]. Findings have shown that the discovery of specific exosomal content and markers may represent a novel diagnostic tool [12, 13]. Since the majority of serum miRNAs are encapsulated in exosomes in order to be more stable, the exosomal miRNAs could be applied as promising non-invasive biomarkers and potential targetable factors in cancer diagnosis and treatment [9, 14, 15]. Recent studies have focused on “tumor-specific” or “tumor-enriched” miRNAs which are specifically packaged into exosomes [16–19].

Exosomal miRNA profiling of serum from different cancer patients versus healthy controls has revealed significant differences in relation to cancer development and metastasis, indicating a possible use of these miRNAs as prognostic biomarkers [20].The role of specific exosomal miRNAs as biomarkers in cancer diagnosis, prognosis, and screening have been studied in several types of cancers, including GI cancers [15–17, 21–32].

Some of the serum exosomal miRNAs can be used as biomarkers to predict the recurrence of GI carcinomas such as hepatocellular cancer and esophageal squamous cell carcinoma [33, 34]. The diagnostic and prognostic roles of exosomal miRNA profiles of colorectal cancer (CRC), as the most common malignancy of the GI system, have been evaluated in several studies [15, 31, 35–40].

Previous studies had been developed to investigate the preclinical and clinical characteristics of GI cancers and have led to improved overall and progression-free survival rates. However, there is an emerging potential for screening, diagnosis, or surveillance of cancer and the designing of new therapeutic approaches, especially in the cases of metastatic GI carcinomas. The identification and validation of biomarkers that can be measured routinely in easily accessible samples, including plasma, and that are able to diagnose cancer and predict treatment efficacy and the risk of progression or relapse is a major challenge in cancer research. To date, many researchers have published their data on the clinical relevance of micoRNAs and exosomal miRNA expression and circulating exosomal miRNAs in different clinical and non-clinical samples including tissue, stool, serum, and cell lines of GI cancers [15, 31, 35, 40–44].

Recently, a published review showed that the exosomal miRNAs could be detected and isolated from body fluids such as saliva. This review primarily provided an overview on exosomal miRNAs as diagnostic markers of GI cancers with a focus on the origin and trafficking of exosomes between cells and techniques to isolate exosomal miRNAs, micoRNAs, and exosomal miRNAs expression [44]. However, there is currently no systematic review on circulating exosomal miRNAs as promising non-invasive biomarkers for GI cancers. In the current systematic review, we focus on studies that have evaluated circulating exosomal miRNAs as non-invasive biomarkers in serum of patients with primary GI tumors. We also aim to carry out a systematic review of published exosomal miRNAs, profiling studies that were performed by microarray and real-time PCR methods and then compare the exosomal miRNA expression profiles between primary and metastatic tumors in the serum or plasma of GI cancer patients. Additionally, we intend to identify the most consistently downregulated and upregulated circulating exosomal microRNAs in GI cancers and introduce some of them as diagnostic and prognostic biomarkers for non-invasive clinical applications. Although identification of exosomal miRNAs in other clinical specimen may be significant, in practice, it is difficult to apply invasive methods on patients. On the other hand, it would be challenging to apply findings about exosomal miRNAs expression in non-clinical samples into clinical contexts. Therefore, this systematic review aims to evaluate the circulating exosomal miRNAs as promising non-invasive biomarkers of GI cancers, for the first time.

## Methods/Design

### Aim of the study

The primary objective of this review is to provide a systematic review of primary studies to estimate the frequency of exosome-encapsulated miRNAs in the circulating blood of patients with GI cancers. The secondary objectives are to identify the expression pattern (up- and downregulation) of exosomal miRNAs in patients with different gastrointestinal tract cancers and to identify the specific circulating exosomal miRNAs involved in the diagnosis and prognosis of GI cancers. This review will complement the findings of an existing review published in 2016 [44].

### Review questions

This review of studies should address the following points:

1. To establish the frequency of circulating exosomal miRNAs in GI cancers.
2. To establish the up- and downregulated circulating exosomal miRNAs in GI cancers.
3. To identify the specific circulating exosomal miRNAs involved in the diagnosis and prognosis of GI cancers.

### Study design

This review protocol has been published in the PROSPERO International prospective register of systematic reviews (http://www.crd.york.ac.uk/PROSPERO), with the registration number CRD 42017057129. In addition, the completed review will be reported in line with the Preferred Reporting Items for Systematic Reviews and Meta-Analyses (PRISMA) guidance [45] (Additional file 1).

### Criteria for considering studies for the review

#### Inclusion criteria

Observational studies (cross-sectional, case-control, and cohort studies) investigating circulating exosomal miRNAs in diagnosis and prognosis of GI malignancies, including upper and lower GI, and hepatopancreato-biliary will be included.

### Participants

The participants are patients with GI cancers.

### Types of studies to be excluded

Studies assessing in vitro and in vivo models, reviews, letters, editorials, case reports, and case series studies will be excluded.

### Search methods for identification of studies

#### Electronic searches

The search strategy for identification of relevant studies will comprise the following main electronic databases: PubMed, Web of Science, Embase, and Scopus, as well as Google Scholar search engines with no language restrictions (up to July 2017). An example of the PubMed search strategy is shown in Table 1. The search syntax will be modified in other databases accordingly.

**Table 1:** Search strategy for PubMed

Quary	Search syntax
	Related terms/synonyms
#1	(mesentery[Mesh] OR stomach[Mesh] OR Pancreas[Mesh] OR rectum[Mesh] OR duodenum[Mesh] OR jejunum[Mesh] OR ileum[Mesh] OR cecum[Mesh] OR colon[Mesh] OR mouth[Mesh] OR esophagus[Mesh] OR Gastrointestinal Tracts[Mesh] OR “Gastrointestinal Tracts”[tiab] OR “GI Tract”[tiab] OR “GI Tracts”[tiab] OR “gastrointestinal tract”[tiab] OR “Gastrointestinal Cancer”[tiab] OR “Digestive Tract”[tiab] OR “Digestive Tracts”[tiab] OR “Lower GI Tract”[tiab] OR GI[tiab] OR oral[tiab] OR mouth[tiab] OR esophagus[tiab] OR gullet[tiab] OR gastric[tiab] OR duodenum[tiab] OR jejunum[tiab] OR ileum[tiab] OR cecum[tiab] OR colon[tiab] OR colorectal[tiab] OR sigmoid[tiab] OR rectum[tiab] OR anus[tiab] OR mesentery[tiab] OR hepatic[tiab] OR liver[tiab] OR hepatocellular[tiab] OR stomach[tiab] OR Pancrea*[tiab])
#2	(Neoplasms [Mesh] OR Cancer*[tiab] OR Neoplasm*[tiab] OR Carcinoma[tiab] OR Tumo*[tiab])
#3	(extracellular vesicles[Mesh] OR exosomes[Mesh] OR exosome*[tiab] OR “extracellular vesicle”[tiab] OR “extracellular vesicles”[tiab] OR microvesicle[tiab] OR “Shedding Microvesicles”[tiab])
#4	#1 AND #2 AND #3

The asterisk (*) is used to signify truncation

### Other resources

Reference lists of relevant primary studies, reviews, and key journals will be searched for additional studies.

### Screening of the studies

Duplicate studies will be removed. All databases will be searched, and the titles and abstracts will be extracted (EGh). All the retrieved titles and abstracts from the electronic search will be screened, and the relevant data will be independently extracted (EGh and AR) according to the previously described inclusion criteria. The extracted data will then be cross-checked to rule out any discrepancies. Unresolved discrepancies will be referred to a third author to solve (LJ). All the reasons for exclusion of ineligible studies will be recorded.

### Data extraction (selection and coding)

Data for each of the included studies will be extracted as follows:

1. Study characteristics (first author’s surname, publication year, publication language, country, study design, setting, locations, criteria for sample selection and sample size, diagnostic criteria, outcomes measured, patient enrolment strategies, exosome isolation and identification methods, miRNA profiling, real-time PCR results of serum samples)
2. Participants’ characteristics (age, gender)
3. Frequency estimates of expression of circulating exosomal miRNAs

Discrepancies between two reviewers will be resolved by consensus. An independent investigator will be consulted through discussion to reach consensus where there is uncertainty or disagreement between reviewers.

Corresponding authors will be contacted if further information is needed. If no response is received after sending a reminder, studies will be excluded.

### Risk of bias (quality) assessment

Quality assessment of observational studies is challenging due to their methodological complexities and the subjective nature of quality evaluations [46]. The Cochrane Collaboration endorsed the use of the Newcastle-Ottawa Scale (NOS) to assess the quality of observational studies in its 2011 handbook [47, 48]. The final risk of bias of included studies will be performed by the above mentioned tool. Two reviewers will independently assess the methodological quality of primary studies by the NOS. This tool is used for assessing the quality of non-randomized studies included in a systematic review and/or meta-analyses. The method was developed as a collaboration between the University of Newcastle, Australia, and the University of Ottawa, Canada, using a Delphi process to define variables for data extraction. Using the tool, each study is judged on eight items, categorized into three groups: the selection of the study groups, the comparability of the groups, and the ascertainment of either the exposure or outcome of interest for case-control or cohort studies respectively [49]. Separate tools were developed for cohort and case-control studies. It has also been adapted for cross-sectional studies [50–52]. For more details, see three appendix scales for evaluating the quality assessment of the above mentioned observational studies (Additional files 2, 3, and 4).

### Strategy for data synthesis

Given the considerable variability among cancers and the difficulty in detecting common exosomal miRNAs and characteristics of the studies, the extracted outcomes will be summarized and reported using descriptive statistics without conducting any meta-analyses. Two separate tables will be used in all included studies. The first table will provide details on study quality according to the mentioned tool, and the other one will include study design, participants, and frequency of exosomal miRNAs in patients with GI cancers. The publication bias will be assessed by funnel plots (i.e., plots of study results against precision) and Beggs’ and Eggers’ tests. We will assess the overall quality of the evidence for each main outcome based on the GRADE (Grading of Recommendations Assessment, Development and Evaluation) system [53, 54]. GRADE provides explicit criteria that address study design, risk of bias, imprecision, inconsistency, indirectness, and magnitude of the effect to rate the quality of evidence across studies. This method rates the quality of the evidence from high (very confident that the true effect lies close to that of the estimated effect) to very low (very little confidence in the effect estimate).

### Sensitivity analysis

Given the diversity of cancer types and different reported exosomal miRNAs across the studies, we anticipate that we cannot perform a meta-analysis; therefore, no sensitivity analysis will be applicable to this study.

### Analysis of subgroups or subsets

If the data is determined to be heterogeneous, we will conduct a narrative synthesis of the findings from the included studies; therefore, a subgroup analysis will not be applicable to this study.

## Discussion

Emerging evidence supports the hypothesis that exosomal microRNAs can be used as circulating diagnostic biomarkers of several cancers. However, there is currently no collective view of which circulating exosomal microRNAs are suitable candidates. The distinctive properties of miRNAs within exosomes can be manipulated to render them suitable for the development of effective diagnostic and targetable tools for non-invasive screening and targeting of tumor cells in cancer patients. Reviews showing the serum exosomal microRNAs in different cancers have already emerged [1, 39, 44], but to date, this will be the first systematic review to evaluate the circulating exosomal miRNAs in the serum of patients with GI cancers. The findings of this review may be used in clinical settings and in designing and developing of the novel non-invasive biomarkers for early diagnosis and pathological condition of patients with GI cancers.

## Additional files

Additional file 1: PRISMA-P (Preferred Reporting Items for Systematic Review and Meta-Analysis Protocols) 2015 checklist [45]. (DOC 85 kb)

Additional file 2: Newcastle-Ottawa quality assessment scale for cross-sectional studies. (PDF 189 kb)

Additional file 3: Newcastle-Ottawa quality assessment scale for case-control studies. (PDF 17 kb)

Additional file 4: Newcastle-Ottawa quality assessment scale for cohort studies. (PDF 93 kb)

## Abbreviations

GI: Gastrointestinal
microRNAs: miRNAs, miRs
PRISMA: Preferred Reporting Items for Systematic Reviews and Meta-Analyses
