# Supplementary material for: Exosomal microRNAs as potential circulating biomarkers in gastrointestinal tract cancers: a systematic review protocol
Source: Syst Rev. 2017 Nov 17;6:228. doi: 10.1186/s13643-017-0624-2 (PMC5693515; doi:10.1186/s13643-017-0624-2)
Supplement: Supplementary file 4 — Newcastle-Ottawa quality assessment scale for cohort studies. (PDF 93 kb) [file 13643_2017_624_MOESM4_ESM.pdf]

## NEWCASTLE - OTTAWA QUALITY ASSESSMENT SCALE
## COHORT STUDIES

*Note:* *A study can be awarded a maximum of one star for each numbered item within the Selection and Outcome categories. A maximum of two stars can be given for Comparability*

**Selection**

1) Representativeness of the exposed cohort
   a) truly representative of the average ________________ (describe) in the community*****
   b) somewhat representative of the average _______________ in the community ¯
   c) selected group of users eg nurses, volunteers
   d) no description of the derivation of the cohort
2) Selection of the non exposed cohort
   a) drawn from the same community as the exposed cohort*****
   b) drawn from a different source
   c) no description of the derivation of the non exposed cohort
3) Ascertainment of exposure
   a) secure record (e.g, surgical records)*****
   b) structured interview*****
   c) written self report
   d) no description
4) Demonstration that outcome of interest was not present at start of study
   a) yes
   b) no

**Comparability**

1) Comparability of cohorts on the basis of the design or analysis
   a) study controls for ______________ (select the most important factor) *****
   b) study controls for any additional factor***** (This criteria could be modified to indicate specific control for a second important factor.)

**Outcome**

1) Assessment of outcome
   a) independent blind assessment*****
   b) record linkage*****
   c) self report
   d) no description
2) Was follow-up long enough for outcomes to occur
   a) yes (select an adequate follow up period for outcome of interest) *****
   b) no
3) Adequacy of follow up of cohorts
   a) complete follow up - all subjects accounted for*****
   b) subjects lost to follow up unlikely to introduce bias - small number lost - > ____ % (select an adequate %) follow up, or description provided of those lost) *****
   c) follow up rate < ____% (select an adequate %) and no description of those lost
   d) no statement

Wells, G. A, Shea, B., O'Connel, D. et al. The Newcastle-Ottawa scale (NOS) for assessing the quailty of nonrandomised studies in meta-analyses. http://www ohri ca/programs/clinical_epidemiology/oxford htm 2009 Feb 1
